# Cross-Subject Commonality of Emotion Representations in Dorsal Motion-Sensitive Areas

**DOI:** 10.3389/fnins.2020.567797

**Published:** 2020-10-14

**Authors:** Yin Liang, Baolin Liu

**Affiliations:** ^1^Faculty of Information Technology, College of Computer Science and Technology, Beijing Artificial Intelligence Institute, Beijing University of Technology, Beijing, China; ^2^School of Computer and Communication Engineering, University of Science and Technology Beijing, Beijing, China

**Keywords:** functional magnetic resonance imaging, emotion perception, multivariate pattern analysis, motion-sensitive areas, cross-subject decoding

## Abstract

Emotion perception is a crucial question in cognitive neuroscience and the underlying neural substrates have been the subject of intense study. One of our previous studies demonstrated that motion-sensitive areas are involved in the perception of facial expressions. However, it remains unclear whether emotions perceived from whole-person stimuli can be decoded from the motion-sensitive areas. In addition, if emotions are represented in the motion-sensitive areas, we may further ask whether the representations of emotions in the motion-sensitive areas can be shared across individual subjects. To address these questions, this study collected neural images while participants viewed emotions (joy, anger, and fear) from videos of whole-person expressions (contained both face and body parts) in a block-design functional magnetic resonance imaging (fMRI) experiment. Multivariate pattern analysis (MVPA) was conducted to explore the emotion decoding performance in individual-defined dorsal motion-sensitive regions of interest (ROIs). Results revealed that emotions could be successfully decoded from motion-sensitive ROIs with statistically significant classification accuracies for three emotions as well as positive versus negative emotions. Moreover, results from the cross-subject classification analysis showed that a person’s emotion representation could be robustly predicted by others’ emotion representations in motion-sensitive areas. Together, these results reveal that emotions are represented in dorsal motion-sensitive areas and that the representation of emotions is consistent across subjects. Our findings provide new evidence of the involvement of motion-sensitive areas in the emotion decoding, and further suggest that there exists a common emotion code in the motion-sensitive areas across individual subjects.

## Introduction

The ability to understand emotions is a crucial social skill in humans. It has been proposed that body language plays an important role in conveying emotions (Calbi et al., 2017). Body language refers to the non-verbal signals in which physical behaviors, including facial expressions, body posture, gestures, eye movement, touch and the use of space, are used to express our true feelings and emotions. According to experts, these non-verbal signals make up a huge part of our daily communication. Humans can easily recognize others’ emotions from their whole-person expressions and perceive them in a categorical manner. Since the human brain can readily decode emotions, considerable functional magnetic resonance imaging (fMRI) studies have investigated the potential neural substrates and mechanisms underlying the perception of emotions.

Neuroimaging studies on emotion perception have used emotional faces or non-face bodies as stimuli and identified specific areas showing preferential activation patterns, respectively known as face-selective and body-selective areas. Classical face-selective areas mainly contain the fusiform face area (FFA), occipital face area (OFA), and superior temporal sulcus (STS), which are together considered the “core system” in Haxby’s model (Haxby et al., 2000; Kanwisher and Yovel, 2006; Gobbini and Haxby, 2007; Pitcher, 2014; Henriksson et al., 2015). Emotional bodies are found to be represented in the extrastriate body area (EBA) and fusiform body area (FBA), and some similarities have been revealed between the processing of emotional bodies and faces (Minnebusch and Daum, 2009; de Gelder et al., 2010; Kret et al., 2011; Downing and Peelen, 2016). In addition, the STS, which acts as a crucial node for social information processing, has been found to be involved in the processing of emotions in both faces and bodies (Candidi et al., 2011; Zhu et al., 2013). Previous fMRI studies mainly assessed the perception of emotions using either isolated faces or non-face bodies as visual stimuli. However, behavioral studies have indicated that human brain prefers whole-person expressions which contain both the face and body parts, similar to that which we commonly perceive in real scenes, and encoding whole-person expressions in a holistic rather than part-based manner (Soria Bauser and Suchan, 2015). Therefore, it is essential to explore the neural representation of whole-person expressions individually rather than in an integrated manner based on the isolated emotional faces and bodies (Zhang et al., 2012; Soria Bauser and Suchan, 2015). Moreover, most previous studies used static emotional images as stimuli, but, considering that the emotions we mostly encounter in a natural context are dynamic, recent studies have proposed that dynamic stimuli are more ecologically valid than their static counterparts (Johnston et al., 2013; Yang et al., 2018). Thus, using dynamic emotional stimuli may be more appropriate to investigate the authentic mechanisms used to recognize emotions in daily life.

Compared to univariate analyses that estimate emotion-evoked responses, a multivariate pattern analysis (MVPA), as demonstrated by recent fMRI studies, can take advantage of distributed activation patterns in fMRI data, thus providing a more effective method to infer the functional roles of cortical regions in emotion perception (Mahmoudi et al., 2012). A growing number of studies have used ROI-based MVPA to explore emotion decoding performances in specific brain areas (Said et al., 2010; Harry et al., 2013; Wegrzyn et al., 2015). In addition, studies with dynamic stimuli have found that dorsal motion-sensitive areas within human motion complex (hMT) + /V5 and STS exhibited significant responses to facial expressions (Furl et al., 2013, 2015). A macaque study identified motion-sensitive areas in the STS, which may be homologous to human STS, and found that facial expressions could be successfully decoded from motion-sensitive areas (Furl et al., 2012). Moreover, one of our recent studies has also identified the successful decoding of dynamic facial expressions in motion-sensitive areas (Liang et al., 2017). These findings suggest that motion-sensitive areas may transmit measurable quantities of expression information and may play an important role in emotion perception. However, these studies only used facial expressions as stimuli, and the full role of motion-sensitive areas in the decoding of whole-person expressions therefore remains unclear. Since we commonly perceive emotions from whole-person expressions in our daily lives, exploring the decoding performance of whole-person expressions in motion-sensitive areas may be meaningful in revealing the potential mechanisms by which the human brain efficiently recognizes emotions from body movements. Furthermore, if emotions are represented in the motion-sensitive areas, we may further ask whether emotion codes in the motion-sensitive areas can be shared across individual subjects. This would shed light on whether an individual’s subjective emotion representation in motion-sensitive areas corresponds to those observed in others, which would be helpful in assessing the commonality and variability of emotion coding.

In this study, we conducted a regions of interest (ROI) MVPA to assess the potential role of dorsal motion-sensitive areas in emotion decoding. We performed a block-design fMRI experiment and collected neural images while participants viewed emotional videos expressed by whole-person expressions (joy, anger, and fear). Dynamic emotion stimuli were used in this study to enhance ecological validity and to assess the authentic mechanisms of emotion recognition in daily life. A separate localizer was used to identify individual-defined motion-sensitive ROIs. We first examined whether emotions could be decoded based on the activation patterns from motion-sensitive ROIs, after which we examined whether there exists a common representation of emotions in motion-sensitive areas across individuals.

## Materials and Methods

### Participants

A total of 24 healthy, righted-handed college students participated in the experiment (12 males, ranging from 19–25-years-old). All subjects had normal or corrected-to-normal vision, with no history of neurological disorders, and signed informed written consent forms before the experiment. Experimental procedures were explained to them before the scanning. The threshold for head motion was framewise displacement (FD) < 0.5 mm (Power et al., 2012). Four subjects were discarded due to excessive head motion, and the final fMRI analysis was focused on the data of 20 subjects (10 males, mean age 21.8 ± 1.83 years old). This experiment was approved by the local Ethics Committee of Yantai Affiliated Hospital of Binzhou Medical University. A separate group of subjects (*n* = 18, 8 females, mean age: 22.2 years old) participated in a preliminary behavioral experiment for the stimulus validation.

### Experimental Procedures

The fMRI experiment was based on a block design, with four “main experiment” runs for the emotion perception task and one “localizer” run for the ROI identification. A separate localizer was used in our study to ensure that the data used for the ROI definition was independent of the data used for the classification in the main experiment analysis (Axelrod and Yovel, 2012, 2015; Furl et al., 2013; Harry et al., 2013).

**FIGURE 1:**
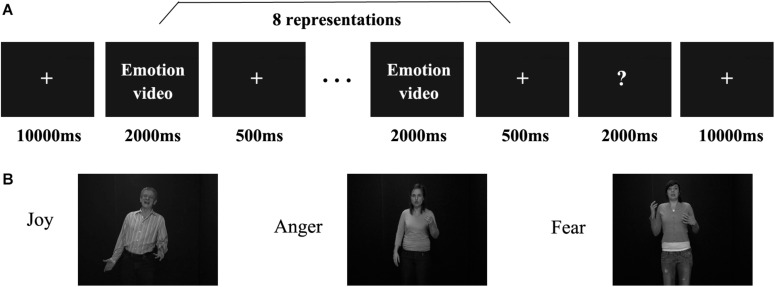
Paradigm representation of the main experiment and example emotional stimuli. **(A)** Schematic representation of the paradigm used. A cross was presented for 10 s before each block, after which eight emotional stimuli appeared. Subsequently, participants completed a button task to indicate their identification of the emotion category they had seen in the previous block. **(B)** All emotional stimuli were taken from the geneva multimodal emotion portrayals (GEMEP) database. Videos of whole individuals displaying three emotions (joy, anger, and fear) were used in the experiment.

Figure 1A shows the process of the main experiment. Each run began with a 10 s fixation cross followed by 18 stimulus blocks presented in a pseudo-random order (Axelrod and Yovel, 2012; Furl et al., 2013, 2015). Successive stimulus blocks were separated by 10 s intervals of a fixation cross. For the first three runs, three emotions (joy, anger, and fear) expressed by three stimulus types (facial, non-face bodily, and whole-person stimuli) were presented in different blocks, while for the fourth run, only three emotions expressed by the whole-person stimuli were presented. In each block, eight video clips of different examples per emotion category were displayed (each for 2000 ms), with an interstimulus interval (ISI) of 500 ms. At the end of each block, there was a 2 s button task instructing participants to indicate the emotion category they had seen by pressing a button. The emotion stimuli were taken from the geneva multimodal emotion portrayals (GEMEP) corpus (Banziger et al., 2012). Videos of eight individuals (four males and four females) displaying three emotions (joy, anger, and fear) were selected as whole-person emotion stimuli (Cao et al., 2018; Yang et al., 2018). Facial and bodily emotion stimuli were generated from the whole-person videos by cutting out and obscuring the irrelevant part with Gaussian blur masks using Adobe Premiere Pro CC (Kret et al., 2011). All video clips were cropped to 2,000 ms (25 frames/s) to retain the transition from a neutral expression to the emotion apex, and were converted into grayscale using MATLAB (Furl et al., 2012, 2013, 2015; Kaiser et al., 2014; Soria Bauser and Suchan, 2015). The resulting videos were resized to 720 × 576 pixels and presented on the center of the screen. All generated emotion stimuli were validated by another group of participants before scanning, confirming the validity of the stimuli in representing all expressions. Figure 1B shows the examples of whole-person emotion stimuli in the main experiment.

In the functional localizer run, participants viewed video clips or static images of four categories: faces, non-face bodies, whole-persons and objects. Each category appeared two times in a pseudo-random order, resulting in 16 blocks in total (4 categories × video/image × 2 repetitions). Each block contained 8 stimuli (7 novel and 1 repeated), and each was presented for 1,400ms, separated by an ISI of 100 ms. Participants performed a “one-back” task during the localizer run, that is, to press a button when they observed two identical stimuli appearing in consecutive trials.

The stimuli were presented using E-Prime 2.0 Professional (Psychology Software Tools, Pittsburgh, PA, United States) and the behavioral results were collected using the response pad in the scanner. After scanning, participants were required to complete a questionnaire recording whether participants performed the experiment according to the instructions, their feelings during the fMRI experiment, and any difficulties they encountered.

### Data Acquisition

Imaging data were acquired from Yantai Affiliated Hospital of Binzhou Medical University, using a 3.0-T SIEMENS MRI scanner with an eight-channel head coil. Acquisition parameters of task-related functional images and anatomical images were as follows: T2^∗^-weighted functional images were collected using a gradient echo-planar imaging (EPI) sequence, with repetition time (TR) = 2,000 ms, echo time (TE) = 30 ms, voxel size = 3.1 mm × 3.1 mm × 4.0 mm, matrix size = 64 × 64, slices = 33, slices thickness = 4 mm, slice gap = 0.6 mm (Yang et al., 2018). T1-weighted anatomical images were acquired using a three-dimensional magnetization-prepared rapid-acquisition gradient echo (3D MPRAGE) sequence, with TR = 1,900 ms, TE = 2.52 ms, time of inversion (TI) = 1100 ms, voxel size = 1 mm × 1 mm × 1 mm, matrix size = 256 × 256. Participants viewed the emotion stimuli through the high-resolution stereo 3D glasses of the VisuaStim Digital MRI Compatible fMRI system. Foam pads and earplugs were used during scanning to reduce head motion and scanner noise.

### Preprocessing

Statistical parametric mapping 8 (SPM8) software^1^ was used to preprocess the functional and structural images. For each functional run, the first five volumes were discarded to minimize the magnetic saturation effect. Slice-timing and head motion correction were performed for the remaining functional images. The threshold for head motion was FD < 0.5 mm (Power et al., 2012). Next, the structural images were co-registered to the mean functional image after motion correction, and were then unified segmented into gray matter, white matter (WM) and cerebrospinal fluid (CSF). The functional data were spatially normalized to the standard Montreal Neurological Institute (MNI) space using normalization parameters estimated from the unified segmentation, after which the voxel size was re-sampled into 3 mm × 3 mm × 3 mm. Subsequently, the normalized functional images of the localizer run were spatially smoothed with a 6-mm full-width at half-maximum Gaussian kernel to improve the signal-to-noise ratio.

### Localization of Dorsal Motion-Sensitive Regions of Interest (ROIs)

**FIGURE 2:**
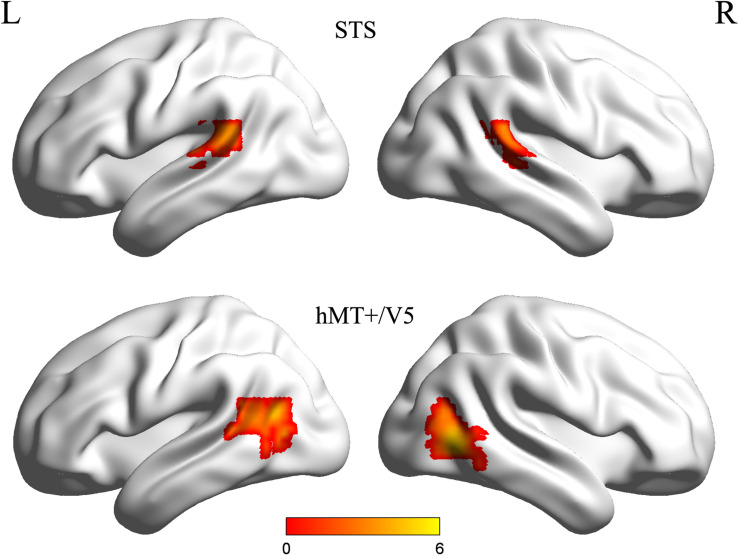
Statistical maps of the significant clusters of superior temporal sulcus (STS) and hMT + /V5 of a representative subject (uncorrected *p* < 0.05 with a cluster size > 20 voxels). Individual subjects’ motion-sensitive regions of interest (ROIs) were defined as 9 mm spheres surrounding the peak coordinates.

Individual ROIs were defined using the localizer run data in which participants viewed static and dynamic faces, non-face bodies, whole persons and objects. At the first-level (within-subject) analysis, a general linear model (GLM) was constructed for each subject to estimate the task effect for each condition: dynamic face, static face, dynamic body, static body, dynamic whole-person, static whole-person, dynamic object and static object. Each regressor was modeled by a boxcar function (representing the onsets and the durations of the stimulus blocks) convolved with a canonical hemodynamic response function (HRF). Several confounding nuisances were regressed out along with their temporal derivatives, including the realignment parameters from head motion correction and the physiological noise from WM and CSF were regressed using the CompCor (Behzadi et al., 2007; Whitfield-Gabrieli and Nieto-Castanon, 2012; Woo et al., 2014; Power et al., 2015; Xu et al., 2017; Geng et al., 2018). The low-frequency drifts of the time series were removed with a 1/128 Hz high-pass filter. The dorsal motion-sensitive ROIs were then identified by contrasting the average response to dynamic versus static conditions. The aim of using this contrast was to identify the motion-sensitive areas which are relatively domain-general, as both person and person parts, as well as those focused on non-person objects. We were especially interested in whether emotions perceived from whole-person expressions could be decoded from the relatively domain-general motion-sensitive areas, which are not specialized for representing only facial or bodily attributes. Thus, we chose to use a contrast which was expected to elicit motion areas to be domain general. Previous studies have showed that combined different types of stimuli together would be expected to localize motion-sensitive responses subsuming areas to be relatively domain-general (Furl et al., 2012, 2013, 2015; Liang et al., 2017). Therefore, to maximize the available data and to identify relatively domain-general motion-sensitive areas, we chose to average the results for ROI definition. We identified bilateral areas within human hMT + /V5 for all twenty subjects and bilateral STS areas for eighteen subjects, with two subjects only demonstrating a unilateral STS area in the left or right hemisphere. The ROIs were generated with a liberal threshold (*p* < 0.05; Skerry and Saxe, 2014; Miao et al., 2018; Yang et al., 2018). Individual subjects’ motion-sensitive ROIs were defined as 9 mm spheres surrounding the peak coordinates. Subsequent emotion classification analyses were carried out based on these individually defined ROIs using the data from the main experiment runs. Table 1 summarizes the average MNI coordinates (mean ± standard deviation SD) for each ROI, and Figure 2 shows the statistical maps of the significant clusters in the ROI definition of a representative subject (uncorrected *p* < 0.05 with a cluster size > 20 voxels).

**TABLE 1:** Localization of motion-sensitive regions of interest (ROIs) used in the decoding analysis of main experiment data.

**Functional ROIs**	**Hemisphere**	**Number of Subjects**	**MNI Coordinates**		
			***x***	***Y***	***z***
STS	L	19	−55±10	−42±5	14±5
	R	19	57±8	−39±8	13±6
hMT + /V5	L	20	−53±5	−67±5	6±6
	R	20	53±6	−64±7	3±5

*Number of subjects in whom the ROIs were localized and the average Montreal Neurological Institute (MNI) coordinates (mean ± standard deviation SD) are reported. STS, superior temporal sulcus.*

### Within-Subject and Cross-Subject Emotion Classifications

**FIGURE 3:**
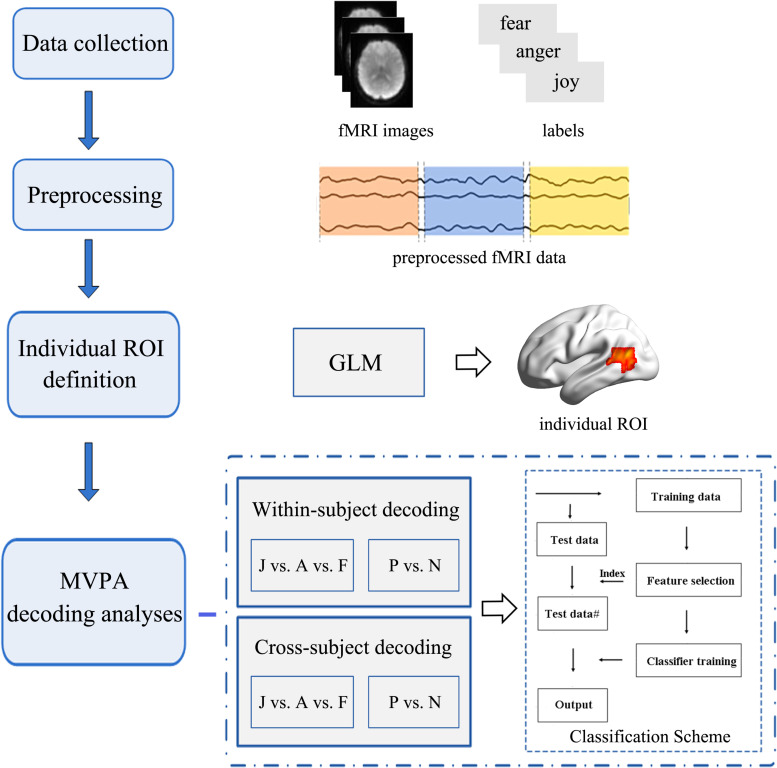
Flowchart of the data analysis procedure. Multivariate pattern analysis (MVPA) emotion decoding analyses were conducted based on the individually defined regions of interest (ROIs). Two types of classification analyses (within-subject classification and cross-subject classification) were performed to examine the potential role of the motion-sensitive ROIs in the emotion decoding. Both three-way (joy vs. anger vs. fear: J vs. A vs. F) and two-way (positive vs. negative: P vs. N) emotion classifications were performed.

Emotion classification analyses were conducted on the unsmoothed data from the main experiment (Harry et al., 2013; Yang et al., 2018) using a MVPA. We carried out MVPA classifications within ROIs that were functionally localized based on individual subject localizer runs. Similar procedures as those in previous MVPA studies were used in this study. For each participant, raw intensity values for all voxels within an ROI were extracted and normalized using the *z*-score function. The MVPA classification was carried out based on the multi-voxel activation patterns. Feature selection was performed using an ANOVA, which yielded a *p*-value for each voxel to tell the probability that a given voxel’s activity varied significantly between emotion conditions. Feature selection was executed only on the training set to avoid peeking, and the threshold for ANOVA was *p* < 0.05. Next, the data were classified using a linear support vector machine (SVM) that was implemented in LIBSVM^2^ (Chang and Lin, 2011; Skerry and Saxe, 2014). The activation patterns for each condition were used to train and test the SVM classifier to perform classification over emotions. Figure 3 represents the framework of our emotion classification analyses. We conducted two types of classifications in this section to assess the potential role of motion-sensitive ROIs in emotion decoding: first, a classical within-subject emotion classification was carried out as implemented in previous MVPA studies (classifier was trained and tested within the same subject data); next, a cross-subject emotion classification was conducted (classifier was trained iteratively on all subjects but one and tested on the remaining one) to assess whether there is any commonality to emotional representations in motion-sensitive areas across individual subjects. The cross-subject classification was performed using a leave-one-subject-out cross-validation (LOOCV) scheme (Chikazoe et al., 2014). In each fold of LOOCV, we trained the classifier in all but one subject and the remaining one was used as the test set. The cross-validation procedure was repeated until each subject was used as the test set, and the classification performance was averaged over all folds. The cross-subject classification was used to further investigate whether emotion codes in the motion-sensitive areas can be shared across subjects.

We ran both three-way (joy vs. anger vs. fear) and two-way (joy versus anger/fear, which could be considered as positive vs. negative) emotion classifications. The three-way classification was implemented similarly as previous MVPA studies (Wang et al., 2016; Liang et al., 2017), using a one-against-one voting strategy. That is, we obtained classifiers for each pair of emotions and these pairwise classifiers were then added to yield the linear ensemble classifier for each emotion. Classifying positive versus negative emotions is essential since these results basically demonstrate coarse-grained emotion codes which can clearly distinguish positive-to-negative valences in bipolar representations, all the while taking into account the fact that some regions may not classify specific emotions in a fine-grained way, but may be able to distinguish positive and negative valence emotion representations (Kim et al., 2017). Data were partitioned into multiple cross-validation folds and the classification accuracies were averaged across folds to yield a single classification accuracy in each ROI. For the within-subject emotion classification, a cross-validation was performed across blocks, while for the cross-subject emotion classification, the cross-validation folds were based on subjects (testing each participant’s activation pattern by a classifier that was trained by all other participants). For the classification of positive versus negative emotions, half of the data from anger and fear conditions were randomly dropped for each cross-validation, equating the base rates and therefore generating a chance level of 0.5 (Kim et al., 2017; Cao et al., 2018). To evaluate the emotion decoding performance, the significance of the classification results was established as a group level one-sample *t*-test above chance level (with a chance of 0.33 for the classification of three emotions, and a chance of 0.5 for the classification of positive versus negative emotions; Wurm and Lingnau, 2015; Cao et al., 2018), and were subsequently corrected for multiple comparisons by false discovery rate (FDR) and Bonferroni corrections according to the number of ROIs.

## Results

### Behavioral Results

Behavioral results of the emotion classification accuracies and the reaction times for each emotion (joy, anger, and fear) are summarized in Table 2. These results confirmed the validity of the emotion stimuli used in our experiment as all emotions were well recognized with a high level of accuracy. Paired *t*-tests for the classification accuracies and reaction times were performed among the three emotions. Results showed that the classification accuracy for joy was significantly higher than that for anger and fear and that there was no significant difference between the accuracies for anger and fear [joy vs. anger: *t*_(__19__)_ = 1.831, *p* = 0.041; joy vs. fear: *t*_(__19__)_ = 2.333, *p* = 0.015; anger vs. fear: *t*_(__19__)_ = 1.286, *p* = 0.107; one-tailed]. For the reaction times, participants showed a significantly quicker response to joy than to anger or fear, and the response time for anger was shorter than that for fear [joy vs. anger: *t*_(__19__)_ = -3.514, *p* = 0.001; joy vs. fear: *t*_(__19__)_ = -6.180, *p* < 0.001; anger vs. fear: *t*_(__19__)_ = -3.161, *p* = 0.003; one-tailed].

**TABLE 2:** Behavioral results (mean % and standard deviations SD).

	**Classification Accuracy (%)**		**Reaction Time (ms)**	
	**Mean**	**SD**	**Mean**	**SD**
Joy	100	0	675.25	155.35
Anger	98.75	3.05	767.05	224.22
Fear	97.08	5.59	836.10	210.54

### Within-Subject and Cross-Subject Emotion Decoding Results

**FIGURE 4:**
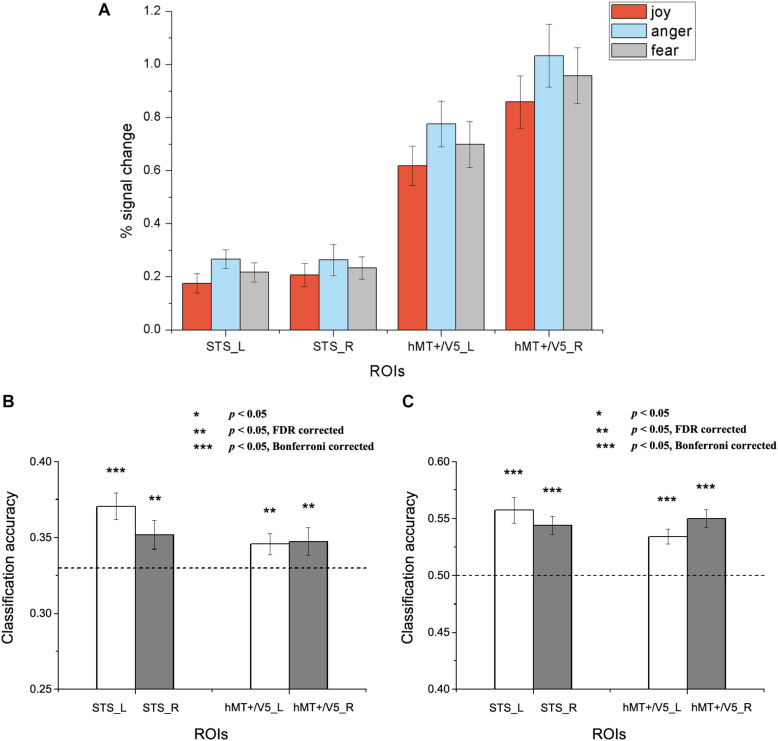
Results of the within-subject emotion decoding analysis. **(A)** Average percent signal change for each emotion, **(B)** Classification accuracies for three emotions, and **(C)** Classification accuracies for positive versus negative emotions. The dashed line indicates chance level, and all error bars represent the standard error of the mean (SEM). Asterisks indicate statistical significance with a one-sample *t*-test, *p* < 0.05 [** *p* < 0.05 false discovery rate (FDR) corrected; *** *p* < 0.05 Bonferroni corrected].

**FIGURE 5:**
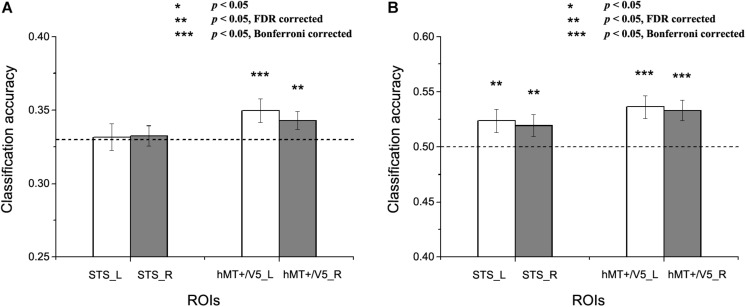
Results of the cross-subject emotion decoding analysis. **(A)** Classification accuracies for three emotions, and **(B)** Classification accuracies for positive versus negative emotions. The dashed line indicates chance level, and all error bars reflect the SEM. Asterisks indicate statistical significance with a one-sample *t*-test, *p* < 0.05 [** *p* < 0.05 false discovery rate (FDR) corrected; *** *p* < 0.05 Bonferroni corrected].

In this section, we conducted MVPA emotion classifications based on the individually defined ROIs. Two types of classification analyses were performed to assess the potential role of the motion-sensitive ROIs in emotion decoding. The first one was a classical within-subject emotion classification which was implemented in a similar way as previous MVPA studies (Axelrod and Yovel, 2012, 2015; Wurm and Lingnau, 2015; Liang et al., 2017). In addition, we conducted a cross-subject emotion classification to assess whether there is any commonality in emotion representations in motion-sensitive areas across individual subjects. Both three-way (joy vs. anger vs. fear) and two-way (joy versus anger/fear, which could be considered as positive vs. negative) emotion classifications were performed. Feature selection was conducted using ANOVA which was executed only on the training data, with a threshold of *p* < 0.05. SVM classifier was trained and tested with cross-validation scheme to perform classification analysis over emotion categories. The classification accuracies for each ROI and subject were entered into one-tailed one-sample *t*-tests against the chance levels (Wurm and Lingnau, 2015), and the statistical results were corrected for multiple comparisons by FDR and Bonferroni corrections according to the number of ROIs. Figures 4, 5 separately show the results for within-subject and cross-subject emotion classifications and the statistical significances for multiple comparisons correction results are indicated by asterisks.

Results for the within-subject emotion decoding analysis are shown in Figure 4, which illustrates the average percent signal change for each emotion (Figure 4A) and the classification accuracies for three emotions (Figure 4B) and for positive versus negative emotions (Figure 4C) in all ROIs. We found that the classification accuracies for three emotions and for positive versus negative emotions were significantly higher than chance in all ROIs [For three emotions classification: left STS: *t*_(__18__)_ = 4.692, *p* < 0.001; right STS: *t*_(__18__)_ = 2.336, *p* = 0.016; left hMT + /V5: *t*_(__19__)_ = 2.294, *p* = 0.018; right hMT + /V5: *t*_(__19__)_ = 1.950, *p* = 0.033. For positive versus negative emotions classification: left STS: *t*_(__18__)_ = 5.149, *p* < 0.001; right STS: *t*_(__18__)_ = 5.478, *p* < 0.001; left hMT + /V5: *t*_(__19__)_ = 5.202, *p* < 0.001; right hMT + /V5: *t*_(__19__)_ = 6.548, *p* < 0.001).

We next assessed whether a person’s emotion representations in the motion-sensitive areas could be predicted by others’ emotion representations. Figure 5 shows the results for the cross-subject emotion classifications in all motion-sensitive ROIs (Figure 5A shows the classification results for three emotions and 5B shows the classification results for positive versus negative emotions). When classifying emotions from the classifiers trained by the activation patterns of other subjects, we found that classification accuracies were significantly higher than chance in hMT + /V5 both for the three emotions and for the positive versus negative emotions [classification of three emotions: left hMT + /V5: *t*_(__19__)_ = 2.483, *p* = 0.01; right hMT + /V5: *t*_(__19__)_ = 2.116, *p* = 0.024; classification of positive vs. negative emotions: left hMT + /V5: *t*_(__19__)_ = 3.510, *p* = 0.001; right hMT + /V5: *t*_(__19__)_ = 3.523, *p* = 0.001]. In the STS, although the classification accuracies for the three emotions did not achieve significance [left STS: *t*_(__18__)_ = 0.174, *p* = 0.432; right STS: *t*_(__18__)_ = 0.351, *p* = 0.365], we did find successful cross-subject positive-to-negative emotion decoding [left STS: *t*_(__18__)_ = 2.199, *p* = 0.021; right STS: *t*_(__18__)_ = 1.995, *p* = 0.031].

## Discussion

In this study, we performed a block-design fMRI experiment and collected neural data while participants viewed emotions (joy, anger, and fear) from videos representing whole-person expressions. Both within-subject and cross-subject MVPA emotion classification analyses were performed to examine the decoding performance of individual-defined motion-sensitive ROIs. We ran both three-way (joy vs. anger vs. fear) and two-way (positive vs. negative) emotion classifications. Our results showed that emotions could be successfully decoded based on the activation patterns in dorsal motion-sensitive areas. Moreover, results from the cross-subject classification analysis showed that motion-sensitive areas supported the classification of individual emotion representation across subjects.

### Emotions Perceived From Whole-Person Expressions Are Represented in Dorsal Motion-Sensitive Areas

We obtained significant classification results for both the classification of the three emotions and the positive versus negative emotions, indicating that emotions perceived from whole-person expressions are represented in the motion-sensitive areas.

Previous studies on facial expressions with dynamic stimuli have revealed a certain degree of sensitivity in dorsal temporal areas, showing that motion-sensitive areas within hMT + /V5 and STS exhibited strong responses to dynamic facial emotions (Foley et al., 2011; Furl et al., 2013, 2015). Considering that the results of the average response from the univariate analysis alone are insufficient to reveal the potential role of a specific brain area underlying decoding (Axelrod and Yovel, 2012; Mahmoudi et al., 2012), recent fMRI studies used ROI-based MVPA to examine the decoding performance of motion-sensitive areas. Furl et al. (2012) used macaque STS as a model system and revealed the successful decoding of facial emotions in motion-sensitive areas. Similar results were obtained in one of our recent studies (Liang et al., 2017). These studies suggest that motion-sensitive areas may transmit measurable quantities of expression information and may be involved in the processing of emotional information. In this study, we defined individual motion-sensitive ROIs and found that emotions perceived from whole-person expressions could be successfully decoded from motion-sensitive areas. Our results are consistent with previous findings, and provide new evidence that emotions perceived from whole-person expressions are represented in the motion-sensitive areas. It should be noted that our results revealed the emotion decoding performance of the relatively domain-general motion-sensitive areas, as the localization contrast we used contained both person and person parts, as well as non-person objects, which was expected to reflect all responses to visual motion (Furl et al., 2012, 2015). Therefore, our results suggest that motion sensitive voxels which respond to various motions, not only specific to facial or bodily attributes, may make a significant contribution to emotion decoding.

Taken together, our findings provide new evidence that emotions are represented in dorsal motion-sensitive areas, pointing to the role of dorsal motion-sensitive areas as key regions in the processing of emotional information in daily communication.

### Commonality of Emotion Representations in Motion-Sensitive Areas Across Individuals

Furthermore, we assessed whether an individual’s emotion representation in the motion-sensitive areas corresponds to that observed in others by conducting a cross-subject emotion classification analysis (classifier was trained iteratively on all subjects but one and tested on the remaining one). This may provide evidence of whether an individual’s subjective emotion representation in the motion-sensitive areas corresponds to that observed in others, which may be helpful in evaluating the commonality and variability in emotion coding (Haxby et al., 2011; Raizada and Connolly, 2012; Chikazoe et al., 2014). We obtained statistically significant results for both the cross-subject classification of three emotions and positive versus negative emotions in the hMT + /V5, indicating that the hMT + /V5 code may reflect experienced emotions in the same way across participants. In addition, although much less significant emotion classification results were identified for the three emotions, we revealed the successful cross-subject classification of positive versus negative emotions in the STS. This reveals that population codes in the STS were less able to decode a specific emotion in a fine-grained way, but demonstrated that the similarity in emotion representations among people may allow for the robust distinction of coarsely defined positive-to-negative emotional valences in the context of bipolar representations (Kim et al., 2017). Our results also suggest that subjective emotion representations are more similarly structured across individual subjects in the hMT + /V5 than in the STS, since hMT + /V5 supported the cross-subject classification of both fine-grained three emotions and coarse-grained positive-to-negative emotions, while the STS only supported the coarse-grained classification in a significant way.

Overall, our study indicates that the representation of emotions in motion-sensitive areas may be similar across participants. This may provide evidence that even in the most subjective perception of an individual’s emotion experience, its internal emotion coding can be predicted on the basis of the patterns observed in others in the motion-sensitive areas. This finding is important, since such cross-subject commonality may allow for the common scaling of the valence of emotional experiences across participants. In summary, we show that a person’s emotional representations in motion-sensitive areas may be predicted by others’ emotional representations, suggesting that there exists a common emotion code in the motion-sensitive areas across individuals.

In the present study, different types of emotional stimuli (facial, bodily, and whole-person expressions) were contained in the main experiment. Future studies with whole-person stimuli separately may further improve the implementation of the classification scheme and lead to better understanding of the whole-person expressions decoding. In addition, compared with ROI-based analyses, whole-brain group-level analyses would provide more informative results. Future studies combine both whole-brain activation-based and FC-based analyses would further enrich our findings about the neural substrates and the mechanisms for the quick and effortless recognition of whole-person emotions.

## Conclusion

Our results showed that emotions perceived from whole-person expressions can be robustly decoded in dorsal motion-sensitive areas. Moreover, successful cross-subject emotion decoding suggests that the emotion representations in motion-sensitive areas could be shared across participants. This study extends previous MVPA studies to the emotion perception of whole-person expressions, which are more frequently perceived in daily life, and may further our understanding of the potential neural substrates underlying the efficient recognition of emotions from body language. Our findings provide new evidence that emotions are represented in dorsal motion-sensitive areas, underscoring the important role of the motion-sensitive areas in the emotion perception. Our study also suggests that emotion representations in motion-sensitive areas are similar across individuals.

## Data Availability Statement

The raw data supporting the conclusions of this article will be made available by the authors, without undue reservation.

## Ethics Statement

The studies involving human participants were reviewed and approved by local Ethics Committee of Yantai Affiliated Hospital of Binzhou Medical University. The patients/participants provided their written informed consent to participate in this study.

## Author Contributions

YL and BL designed the study. YL performed the experiments, analyzed results, and wrote the manuscript. Both authors have approved the final manuscript.

## Conflict of Interest

The authors declare that the research was conducted in the absence of any commercial or financial relationships that could be construed as a potential conflict of interest.

## Footnotes

1 http://www.fil.ion.ucl.ac.uk/spm/software/spm8/

2 http://www.csie.ntu.edu.tw/~cjlin/libsvm/
